# Pursue the truth, devote to education: Chen Hua-Kui, a respectable educator and pioneer of China’s soil microbiology

**DOI:** 10.1007/s13238-016-0270-9

**Published:** 2016-05-05

**Authors:** Doudou Chen, Han Zhang, Huan Liu

**Affiliations:** Wuhan Institute of Virology, Chinese Academy of Sciences, Wuhan, 430071 China

**Figure 1:**
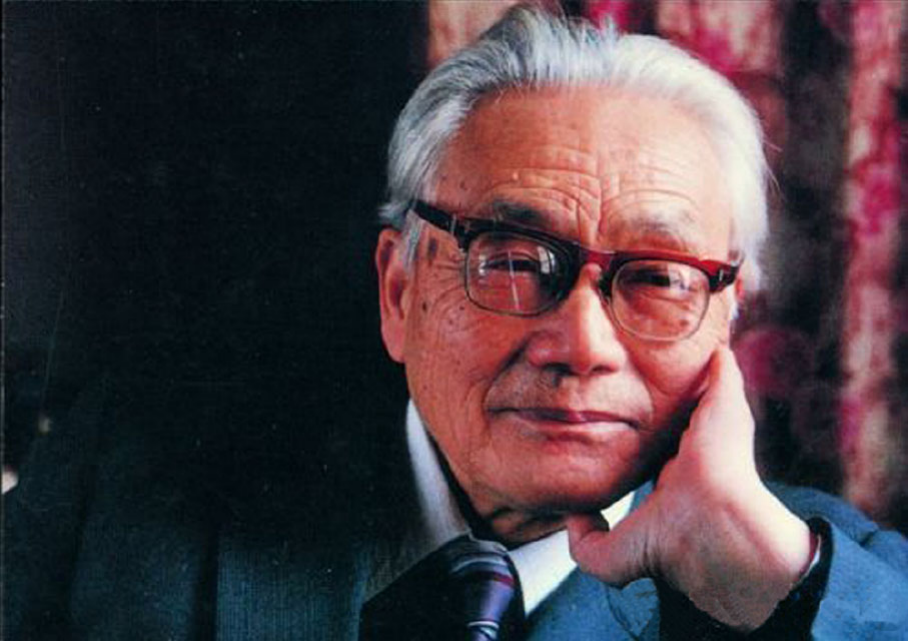
Prof. Chen Hua-Kui (1914–2002)

**Figure 2:**
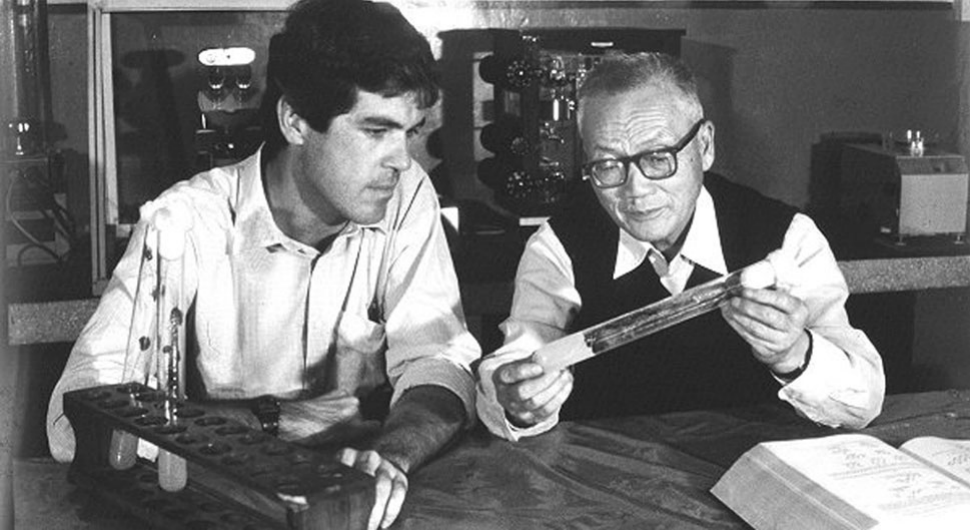
Prof. Chen Hua-Gui and his American student

“Professor Chen Hua-Kui dedicated his whole life to the pursuit of truth, the education of the nation, and services for society. The students of Prof. Chen blossom in the research field.” The Party Committee of Huazhong Agricultural University appraised. Prof. Chen was a renowned microbiologist and distinguished educator in China. In 1944, he first revealed that *Rhizobium astragali* belonged to a select cross-inoculation group, setting the basis for massive production and application of nitragin. Furthermore in 1948, he conducted pioneering research on microbiota and biological cycle of nutrients in wet and dry paddy fields, and in (1964), first discovered the nitrozation of facultatively anaerobic nitrification microorganism in paddy field. In 1980, Prof. Chen was elected as a member of the Academic Divisions of the Chinese Academy of Sciences (CAS) and continued to make great contributions for China’s higher agricultural education and research, especially the research on soil microorganism. (Figs. 1 and 2).

Prof. Chen was born on January 11, 1914 in Beijing. He entered the preparatory class of Peking University in 1928 and graduated from the Biology Department in 1935. After graduation, he taught in the university for one year. In July 1936, with the recommendation of his tutor Zhang Jingyue, he went to England and studied in School of Bacteria & Tropical Medicine, University of London for one year. The following year, Chen began his postgraduate education in the bacteriology laboratory of Rothamsted Experimental Station, England, under the guidance of Dr. Henry Gerard Thornton. There, he undertook research into the symbiotic nitrogen fixation of leguminous plant and rhizobia, and began an over-a-half-century career of soil science and research. Whilst at the Rothamsted Experimental Station, Chen published “*Production of Growth-Substance by Clover Nodule Bacteria*” in *Nature* (Chen, 1938) and his dissertation “*The Structure of* ‘*Ineffective*’ *Nodules and Its Influence on Nitrogen Fixation*” was highly praised by the academic community of England. Chen was awarded a doctor’s degree at the age of 25 in October 1939.

In 1940, during the War of Resistance Against Japanese Aggression, Chen Hua-Kui embarked on a one-year-long arduous journey in the ruins and thorns, returned to China via the United States where he became a researcher in the field of glycolysis in Agricultural Institute led by Prof. Tang Pei-sung in National Southwest Associated University. In 1946, he founded the first Soil Science Department of China in Peking University; in 1947 he founded the Agricultural Chemistry Department in Wuhan University, being a constitution of Huazhong Agricultural University later; in 1956, with Prof. H. Zanyin Gaw, Prof. Chen founded Wuhan Institute of Virology, CAS (Chen et al., 2014).

In the early 1930s, Prof. Chen first discovered that before the root hair of a crop was infected by rhizobia, a hormone secreted by rhizobia was responsible for extending and curling root hair. He conducted comparative research on the morphogenesis of effective and ineffective root nodules, indicating that the amount of nitrogen fixation carried out by a nodulated plant depended on several factors including: the number of nodules, the volume of bacteroid containing tissue in each, the lifecycle of nodule, and the time for which this tissue persisted. He also illustrated the mechanism of symbiotic nitrogen fixation, showing that an effective nodule could fix enough combined nitrogen to nourish the host plant; whereas the ineffective nodule could not (Chen and Thornton 1940).

Prof. Chen investigated the applications of green manure in Yunnan, Sichuan, Shanxi, Guangxi and Hunan Provinces, and conducted researches on the symbiotic nitrogen fixation of *Astragalus* in 1940s. His group isolated for the first time in pure culture the microsymbiont of *Astragalus Sinicus*. In an artificial inoculation test, he discovered that the root-nodule bacteria of *Astragalus* did not produce nodules on other genera of leguminous plants, nor did bacteria isolated from other sources form nodules on *Astragalus* plants, except *Desmodium heterophyllum*, proving that *Astragalus* and its root-nodule bacteria must be considered as a select cross-inoculation group. The research result was published in *Soil Science* in May 1944 (Chen and Shu 1944), which provided the theoretical evidence for large scale artificial inoculation of *Rhizobium astragali*. To commemorate his remarkable contributions, the *International Journal of Systematic Bacteriology* renamed *Rhizobium astragali* into *Mersohizobium huakuii* in 1991.

Prof. Chen was a pioneering researcher of nutrient bio-cycling in paddy fields of China. In 1948, he published a paper on ammoniation of paddy field in both wet and dry seasons along the Yangtze River valley. Ammoniacal nitrogen represents the only source of nitrogen when water is stored in contrast to the use of both nitrate nitrogen and ammoniacal nitrogen when paddy fields are drained in winter for improved aeration. Prof. Chen and his group were the first to discover a sample of anaerobic nitrate bacteria in paddy field, from which they were able to obtain a pure culture, and subsequently reveal the nitrozation effect of facultatively anaerobic nitrifying microorganisms in paddy fields. This discovery was included in the keynote speech *Facultatively Anaerobic Nitrification and Nitrate-forming Organism* delivered on the 8th World Congress of Soil Science (Bucharest) in 1964, and was published on the *Soil Science* (Zhou and Chen 1983), receiving a widespread response in the world.

Prof. Chen was knowledgeable and rigorous in his research and strived to educate his students on the importance of higher education telling them that “Higher education provides you with the hunting gun and method of using it instead of the games.” He earned a great deal of respect from his peers with scientific achievement and moral integrity. Whilst serving as the director of Huazhong Agricultural College, he declined an exclusive car and house instead choosing to continue to live with his wife in an old dormitory of Wuhan University. The Minister of Agriculture Liu Ruilong said that “What precious concern Chen Hua-Kui has for the nation!” Prof. Chen spared no efforts to serve his homeland, and made great contributions for China’s education and research into soil microbiology. His great contributions and virtues will be dearly remembered for generations!
